# Long-term impact of a conditional cash transfer programme on maternal mortality: a nationwide analysis of Brazilian longitudinal data

**DOI:** 10.1186/s12916-021-01994-7

**Published:** 2021-06-01

**Authors:** Davide Rasella, Flávia Jôse Oliveira Alves, Poliana Rebouças, Gabriela Santos de Jesus, Maurício L. Barreto, Tereza Campello, Enny S. Paixao

**Affiliations:** 1 ISGlobal, Hospital Clínic - Universitat de Barcelona, Barcelona, Spain; 2 Institute of Collective Health, Federal University of Bahia (UFBA), Salvador, Brazil; 3 Center of Data and Knowledge Integration for Health (CIDACS), Instituto Gonçalo Muniz, Fundação Oswaldo Cruz (FIOCRUZ), Salvador, Brazil; 4 School of Nutrition, Federal University of Bahia (UFBA), Salvador, Brazil; 5 Future Food Beacon of Excellence, Centre for Research in Race and Rights, School of Biosciences, University of Nottingham, Nottingham, United Kingdom; 6 Faculty of Epidemiology and Population Health, London School of Hygiene and Tropical Medicine, London, United Kingdom

**Keywords:** Conditional cash transfer, Bolsa Família Programme, Maternal mortality

## Abstract

**Background:**

Reducing poverty and improving access to health care are two of the most effective actions to decrease maternal mortality, and conditional cash transfer (CCT) programmes act on both. The aim of this study was to evaluate the effects of one of the world’s largest CCT (the Brazilian Bolsa Familia Programme (BFP)) on maternal mortality during a period of 11 years.

**Methods:**

The study had an ecological longitudinal design and used all 2548 Brazilian municipalities with vital statistics of adequate quality during 2004–2014. BFP municipal coverage was classified into four levels, from low to consolidated, and its duration effects were measured using the average municipal coverage of previous years. We used negative binomial multivariable regression models with fixed-effects specifications, adjusted for all relevant demographic, socioeconomic, and healthcare variables.

**Results:**

BFP was significantly associated with reductions of maternal mortality proportionally to its levels of coverage and years of implementation, with a rate ratio (RR) reaching 0.88 (95%CI 0.81–0.95), 0.84 (0.75–0.96) and 0.83 (0.71–0.99) for intermediate, high and consolidated BFP coverage over the previous 11 years. The BFP duration effect was stronger among young mothers (RR 0.77; 95%CI 0.67–0.96). BFP was also associated with reductions in the proportion of pregnant women with no prenatal visits (RR 0.73; 95%CI 0.69–0.77), reductions in hospital case-fatality rate for delivery (RR 0.78; 95%CI 0.66–0.94) and increases in the proportion of deliveries in hospital (RR 1.05; 95%CI 1.04–1.07).

**Conclusion:**

Our findings show that a consolidated and durable CCT coverage could decrease maternal mortality, and these long-term effects are stronger among poor mothers exposed to CCT during their childhood and adolescence, suggesting a CCT inter-generational effect. Sustained CCT coverage could reduce health inequalities and contribute to the achievement of the Sustainable Development Goal 3.1, and should be preserved during the current global economic crisis due to the COVID-19 pandemic.

**Supplementary Information:**

The online version contains supplementary material available at 10.1186/s12916-021-01994-7.

## Background

Globally, maternal mortality is an important public health problem, and despite strong international efforts, the number of maternal deaths remains high: in 2017 295,000 deaths from maternal causes were estimated worldwide [1].

The association between poverty and maternal deaths has been widely documented, and the majority of these deaths occur in low- and middle-income countries (LMICs), especially those with the largest social inequalities and the lowest income [1, 2]. Poverty affects many factors associated with maternal mortality, such as poor access to public services and infrastructure, unsanitary environmental surroundings, illiteracy, social exclusion, low household income levels and food insecurity [3].

Social protection interventions that aim to reduce social and economic risk, such as conditional cash transfers (CCT), might improve the determinants of maternal health. As a matter of fact, CCT could reduce poverty and improve access to health care services, both priority actions to decrease maternal mortality [4]. Although there is a relative consensus about the impact of CCT on the increase of health care utilisation—contributing to the increase of prenatal and postnatal care visits and to the seeking for skilled delivery [5]—studies on maternal mortality have shown mixed results, with some evaluation indicating significant reduction effects and others showing no impact [4, 6]. Moreover, the evidence of the effects of CCT on maternal deaths is still limited and primarily based on studies that analyse a short period of CCT implementation [4].

Established in 2004, the Brazilian Bolsa Familia Programme (BFP) is one of the world's largest CCT, with the aim of attenuating the effects of poverty through a minimum cash transfer for beneficiary families [7]. The BFP was shown to reduce child mortality and poverty-related infectious diseases [8–10]; however, there are no evaluations of its effects on maternal outcomes. Brazil has also been implemented one of the most effective Primary Health Care strategies in the LMICs, the Family Health Programme [11], that is responsible for the compliance of the BFP conditionalities in health—such as the attendance of prenatal care visits for pregnant women—and has shown synergistic effects with CCT on child health outcomes [12].

Evaluations of the health effects of CCTs in LMIC are particularly relevant during the current—and probably long-lasting—global economic crisis due to the COVID-19, because these poverty-relief interventions could represent an important resilience factor for the most impoverished populations [13, 14].

The objective of this study was to assess the impact of the BFP on maternal mortality in Brazil, also evaluating its effects on potential intermediate mechanisms.

## Methods

### Study design

This study has an ecologic longitudinal design, using municipality as the unit of analysis. We created a joint longitudinal dataset from health, demographic and socioeconomic datasets from 2004 to 2014. More recent years were excluded because of the strong economic crisis which hit the country in 2015, affecting the most vulnerable segments of the society until the current years [15]. While it would have been valuable to include the post-crisis period in our evaluation, no accurate imputation methods were available to estimate municipal-level data for important socioeconomic variables.

From the 5570 Brazilian municipalities, we selected a subset with adequate vital statistics (death and live birth registration)—using a validated multidimensional criterio n[16]—during the first years of the study period (2004–2006), and we assumed constant adequacy for the remaining years due to the documented improvements in vital statistics [17]. The selection of municipalities with adequate vital statistics has been used in similar studies due to the gradual reduction of death underreporting in some of the country’s poorest areas [8]. Such reduction produces an increase of notified deaths (but not necessarily real deaths) that could bias the association between exposure to CCT and mortality rates, underestimating CCT effects.

### Data sources

The data used in this study were collected from different information systems. Mortality Information System for maternal and under-5 deaths, Primary Care Information System for FHP coverage, Live Births Information System and Outpatient Information System for hospital admissions [18]. The Ministry of Social Development databases were used to calculate BFP coverage [19], and the Brazilian Institute of Geography and Statistics (IBGE) was used for socioeconomic variables [20]. Annual values of the socioeconomic covariates obtained from the 2000 and 2010 IBGE National Censuses (income per person, poverty rate, illiteracy, households with piped water) were calculated for the other years, as in previous studies [8–10], through linear interpolation and extrapolation.

### Statistical analyses

We employed multivariable negative binomial regression models for panel data with fixed effects (FE) specifications. Negative binomial regressions are used when the outcome to be analysed is a count (such as deaths during a calendar year), and the Poisson model assumes that the mean is equal to variance is not met, usually because the data present greater dispersion (overdispersion), as in mortality rates [21]. FE models, as any other longitudinal or panel data models, include a second term to control for characteristics of the unit of analysis that are constant during the study period and that have not been included in the model as confounding variables, such as some geographical, historical or sociocultural aspects of each municipality. FE model specification was chosen based on the Hausman test and because it is the most appropriate for impact evaluations with panel data [22]. We used categorised variables for comparison reasons with previous studies [8–10, 23].

We obtained the maternal mortality ratios by direct calculation, expressed as the number of maternal deaths per 100,000 live births. Maternal deaths are identified as the death of a woman while pregnant or within 42 days of the termination of pregnancy, irrespective of the duration and site of the pregnancy, from any cause related to or aggravated by the pregnancy or its management, but not from accidental or incidental causes [1, 24]. As an exposure variable, we used the BFP municipal coverage, which is able to capture not only BFP direct effects on beneficiaries, but also programme externalities (i.e., positive spillover effects on inhabitants not receiving BFP money allowances) [8]. The main indicator of BFP coverage was created in two steps: first, we obtained the annual BFP coverage, calculated as the number of individuals enrolled in the BFP (estimated by multiplying the number of beneficiary families by the average family size) divided by the total population of the same municipality; and second, in order to evaluate BFP duration effects, we calculated the average municipal BFP coverage of the last *n* years (from 1 to 11, the maximum allowed by the study period) for each year under consideration. The average coverage of the last *n* years is obtained, for a given year *y*, by the sum of all yearly coverage of the previous *n*-1 years, plus the coverage of the given year *y*, divided by *n.* This coverage indicator has been used in previous studies to capture the intensity of an intervention coverage along the years of its implementation [23]. The average BFP municipal coverage of the last *n* years was categorised in quartiles representing the levels of BFP implementation over the period, as already done in previous studies [8], low (1st quartile), intermediate (2nd), high (3rd) and consolidated (4th).

As an indicator of Primary Health Care (PHC), we used the average municipal coverage of the last 5 years of the Family Health Programme (FHP), obtained dividing the number of persons into the FHP catchment areas by the total population of the same municipality for each year, and calculating its average in the previous 5 years. This coverage indicator was categorised, for comparability reasons with BFP and other studies [8, 23], in quartiles, from baseline to high. While our study focused on the duration effects of BFP, evaluating how the different years of average BFP coverage were able to affect our outcomes, the years of average FHP coverage were fixed at half of the study period based on previous studies [23], alternative durations were also tested (Additional file 1).

We adjusted the regression model for all relevant covariates recognised as determinants of maternal mortality in the literature [1, 2, 5, 24]. These covariates were dichotomised according to their median value along the period: monthly income per person (median 537.4 Brazilian Reais), poverty rate of the municipality (median 13.5%), proportion of illiteracy among individuals older than 15 years (median 11.1%), percentage of individuals living in households with piped water (median 94.4%), number of physicians per inhabitants (median 0.55), private health care coverage (median 4.46%) and the overall rate of admissions to hospital in the municipality per 1,000 inhabitants (median 3.85). All models include the time variable expressed as a dummy variable, which is the most robust and conservative approach when the outcome is trending and fluctuating over the period [22].

In order to elucidate the causal pathway of BFP effectiveness, we evaluated the association with the proportion of pregnant women with no prenatal visits at the moment of delivery (number of pregnant women who have not done any prenatal visit until the moment of delivery divided by the total number of live births), as well as the effect of BFP on the proportion of deliveries in a hospital (the number of hospitalisations for delivery divided by the total number of live births), and on the hospital case-fatality rate due to delivery (the number of deaths in a hospital due to delivery-associated causes divided by the total number of hospitalisations for delivery).

### Sensitivity analyses

Two negative control outcomes were included in the study: the first was the overall under-five mortality rates (U5MR—number of deaths of children younger than 5 years per 1000 live births) that was used to verify the ability of the models to disentangle the BFP duration effect (BFP should show effects on U5MR since the first year of exposure according to the literature) [8]. The second was U5MR due to accidents (ICD-10 codes V01-X59) that allows to prove the specificity of BFP effects on the selected outcome (BFP has already been shown to have no effects on this group of mortality) [8].

As described in Additional file 1, a wide range of other sensitivity analyses were performed, since the fitting of Poisson—instead of Negative Binomial—multivariable regressions to the use of several alternative indicators of BFP coverage, also including lag effects. All sensitivity analyses were consistent with the results and demonstrated the robustness of the results.

For database processing and analysis, Stata software (version 14.0) was used.

## Results

The criteria for adequate death and live birth registration were met by 2548 municipalities. The mean maternal mortality ratio had fluctuations in the selected municipalities along the study period and decreased over the period (Table 1). Mean BFP municipal coverage increased, reaching 26% in 2014. Socioeconomic conditions improved during the study period, with the mean monthly income per capita increasing by 37.1% and the poverty rate decreasing by 58.7%. Healthcare-related variables, such as the number of physicians and private care coverage, also improved during this period.

**Table 1:** Mean values and SD of the selected variables for the Brazilian municipalities (*n*, 2548)

	Mean (SD) 2004	Mean (SD) 2014	Percentage of change (2004–2014)
PBF coverage	17.83 (12.54)	25.88 (18.87)	45.15
FHP coverage	61.77 (36.87)	70.99 (33.37)	14.93
Income per capita	455.44 (195.21)	624.18 (253.11)	37.05
Poverty rate	27.49 (18.18)	11.37 (13.65)	− 58.64
Illiteracy rate	16.16 (9.95)	12.09 (8.23)	− 25.19
Piped water	78.81 (21.67)	92.53 (13.37%)	17.41
Physicians/hab	0.71 (0.66)	0.80 (0.69)	12.68
Private care coverage	6.15 (8.96)	10.73 (11.62)	74.47
Hospitalisation rates	4.98 (4.47)	3.56 (4.32)	− 28.51
Maternal mortality ratio	60.21 (314.15)	57.89 (308.19)	− 3.85

Table 2 shows the adjusted associations of BFP average coverage with different durations and levels. BFP coverage with limited duration had no statistically significant association with maternal mortality, but with increasing years of duration and increasing levels of municipal coverage, a dose-response relationship was clearly shown. Considering the average BFP coverage duration of 5 years, increasing levels of coverage—medium, high and consolidated—were associated with a maternal mortality reduction, expressed as rate ratio (RR), of 0.91 (95%CI 0.83–0.99), 0.87 (0.77–0.99) and 0.85 (0.72–1.00), respectively. With the duration of 11 years, the same levels of coverage were associated with RR of 0.88 (95%CI 0.81–0.95), 0.84 (0.75–0.96) and 0.83 (0.71–0.99). Models with all range of years of duration are available in Additional file 1. All models are adjusted for all relevant socioeconomic and demographic covariates and include the time variable expressed as a dummy variable.

**Table 2:** Fixed-effect models for adjusted associations between average BFP coverage of the last 1, 2, 5, 10 and 11 years—divided in quartiles—and maternal mortality ratio in the municipalities selected (*n*, 2548) for the quality of vital information in Brazil 2004–2014

Variables	Average BFP coverage 1 year		Average BFP coverage 2 years		Average BFP coverage 5 years		Average BFP coverage 10 years		Average BFP coverage 11 years	
	RR	95% CI	RR	95% CI	RR	95% CI	RR	95% CI	RR	95% CI
BFP low	1	–	1	–	1	–	1	–	1	–
BFP intermediate	0.94	(0.85, 1.05)	0.99	(0.89, 1.11)	0.91	(0.83, 0.99)	0.87	(0.80, 0.95)	0.88	(0.81, 0.95)
BFP high	1.01	(0.88, 1.16)	0.99	(0.87, 1.13)	0.87	(0.77, 0.99)	0.84	(0.75, 0.96)	0.84	(0.75, 0.96)
BFP consolidated	0.94	(0.77, 1.15)	1.03	(0.85, 1.24)	0.85	(0.72, 1.00)	0.82	(0.69, 0.97)	0.83	(0.71, 0.99)
FHP baseline	1	–	1	–	1	–	1	–	1	–
FHP low	0.97	(0.90, 1.06)	0.97	(0.89, 1.06)	0.99	(0.91, 1.08)	0.99	(0.91, 1.08)	0.99	(0.91, 1.08)
FHP intermediate	0.97	(0.86, 1.10)	0.96	(0.84, 1.09)	1	(0.88, 1.14)	0.99	(0.87, 1.13)	0.99	(0.86, 1.13)
FHP high	0.93	(0.79, 1.08)	0.91	(0.78, 1.07)	0.95	(0.81, 1.12)	0.94	(0.79, 1.11)	0.93	(0.79, 1.10)
Income per capita	0.84	(0.72, 0.98)	0.84	(0.72, 0.98)	0.85	(0.72, 0.99)	0.85	(0.72, 0.99)	0.85	(0.72, 0.99)
Poverty rate	1.01	(0.91, 1.12)	0.99	(0.89, 1.09)	0.97	(0.88, 1.08)	0.98	(0.88, 1.09)	0.98	(0.89, 1.09)
Illiteracy rate	0.96	(0.81, 1.13)	0.96	(0.81, 1.13)	0.95	(0.81, 1.12)	0.95	(0.81, 1.13)	0.95	(0.81, 1.13)
Piped water	1.04	(0.93, 1.16)	1.04	(0.93, 1.16)	1.04	(0.93, 1.16)	1.04	(0.93, 1.16)	1.04	(0.93, 1.16)
Physicians/inhab	1.01	(0.91, 1.11)	1.01	(0.91, 1.11)	1.00	(0.91, 1.11)	1.00	(0.91, 1.10)	1.00	(0.91, 1.10)
Private care coverage	0.99	(0.84, 1.17)	0.99	(0.84, 1.17)	0.99	(0.84, 1.17)	0.99	(0.84, 1.17)	0.99	(0.84, 1.17)
Hospitalisation rates	0.91	(0.71, 1.15)	0.91	(0.71, 1.16)	0.91	(0.72, 1.16)	0.91	(0.72, 1.16)	0.91	(0.72, 1.16)
Year^a^	-		-		-		-		-	
No. of observations	17,501		17,501		17,501		17,501		17,501	

^a^The variable year has been used as a categorical dummy variable in the model and its coefficient are shown in Additional file 1

Table 3 shows the effect of BFP on maternal mortality ratios of pregnant women with less than 30 years of age: medium, high and consolidate 11 years BFP duration coverage were associated with a mortality reduction RR of 0.88 (95%CI 0.79–0.99), 0.81 (0.69–0.96) and 0.77 (0.67–0.96), respectively. Two outcomes were used as controls, the first for the duration effects and the second for the levels of coverage: BFP effects on overall under-five mortality rates were present since the first year and were only slightly affected by duration. At the same time, BFP duration and levels of municipal coverage did not affect under-five mortality rate for accidents.

**Table 3:** Fixed effect regression models for adjusted associations between average BFP coverage of the last 1,2,5,10 and 11 years—divided in quartiles—and maternal mortality ratio in women < 30 years in the municipalities selected (n.2548) for the quality of vital information in Brazil 2004–2014. Models are adjusted for the same independent variables of Table 2

Variables	Average BFP coverage 1 year		Average BFP coverage 2 years		Average BFP coverage 5 years		Average BFP coverage 10 years		Average BFP coverage 11 years	
	RR	95% CI	RR	95% CI	RR	95% CI	RR	95% CI	RR	95% CI
**Under-30 maternal mortality**										
BFP low	1	–	1	–	1	–	1	–	1	–
BFP intermediate	0.86	(0.74, 0.98)	0.94	(0.82, 1.08)	1.01	(0.89, 1.14)	0.88	(0.79, 0.98)	0.88	(0.79, 0.99)
BFP high	0.92	(0.78, 1.10)	0.94	(0.79, 1.12)	0.93	(0.79, 1.09)	0.82	(0.69, 0.96)	0.81	(0.69, 0.96)
BFP consolidated	0.81	(0.62, 1.05)	0.89	(0.69, 1.14)	0.93	(0.75, 1.15)	0.75	(0.60, 0.94)	0.77	(0.67, 0.96)
Number of observations	13,288		13,288		13,288		13,288		13,288	
**Under-5 mortality rate**										
BFP low	1	–								
BFP intermediate	1.01	(0.99, 1.03)	1.01	(0.99, 1.03)	0.99	(0.97, 1.01)	0.99	(0.97, 1.00)	0.99	(0.97, 1.00)
BFP high	1.00	(0.97, 1.02)	0.99	(0.97, 1.02)	0.96	(0.94, 0.98)	0.96	(0.94, 0.98)	0.96	(0.93, 0.98)
BFP consolidated	0.95	(0.92, 0.99)	0.93	(0.90, 0.96)	0.92	(0.90, 0.95)	0.91	(0.88, 0.94)	0.91	(0.88, 0.94)
Number of observations	27,973		27,973		27,973		27,973		27,973	
**Under-5 mortality rate from accidents**										
BFP low	1	_	1	_	1	_	1	_	1	_
BFP intermediate	1.18	(1.00, 1.39)	1.13	(0.96, 1.33)	1	(0.86, 1.17)	0.88	(0.76, 1.02)	0.88	(0.76, 1.02)
BFP high	1.02	(0.82, 1.26)	1.11	(0.90, 1.37)	1.12	(0.91, 1.38)	0.95	(0.77, 1.17)	0.92	(0.74, 1.14)
BFP consolidated	0.99	(0.72, 1.38)	1.17	(0.86, 1.59)	1.28	(0.97, 1.69)	1.07	(0.81, 1.43)	1.06	(0.80, 1.41)
Number of observations	12,067		12,067		12,067		12,067		12,067	

The variable year has been used as a categorical dummy in the model and its coefficient are shown in Additional file 1

As shown in Table 4, BFP coverage was negatively associated with the proportion of pregnant women with no prenatal visits at the moment of delivery, reaching a RR of 0.73 (0.69–0.77) for the longest duration and highest BFP coverage level. BFP was also positively associated with an increased proportion of deliveries in hospital, and it was negatively associated with hospital case-fatality rate for delivery, reaching a RR of 0.78 (0.66–0.94) for the longest duration and highest BFP coverage level.

**Table 4:** Fixed-effect models for adjusted associations between average BFP coverage of the last 11 years and percentage of pregnant women with no prenatal visits at the moment of delivery in the municipalities selected (*n*, 2548) for the quality of vital information in Brazil 2004–2014. Models are adjusted for the same independent variables of Table 2

	Proportion of pregnant women with no prenatal visits at the moment of delivery		Proportion of deliveries in hospital		Hospital case-fatality rate for delivery	
	Average BFP cov 11 year		Average BFP cov 11 year		Average BFP cov 11 year	
	RR	CI	RR	CI	RR	CI
BFP low	1	–	1	–	1	–
BFP intermediate	0.92	(0.89, 0.95)	1.04	(1.03, 1.05)	0.83	(0.76, 0.90)
BFP high	0.88	(0.84, 0.92)	1.04	(1.03, 1.05)	0.79	(0.70, 0.90)
BFP consolidated	0.73	(0.69, 0.77)	1.05	(1.04, 1.07)	0.78	(0.66, 0.94)
No. of observations	27,445		28,028		16,683	

## Discussion

This study shows that the exposure to the Brazilian conditional cash transfer Bolsa Familia was associated with reductions in maternal mortality rates, indicating a dose-response relationship with its levels of coverage and years of implementation. This effect was stronger on young mothers, suggesting that exposure to BFP early in life—during childhood and adolescence—could reduce maternal mortality in adult life and, consequently, have an inter-generational effect on the newborns.

BFP was also associated with reductions in the proportion of pregnant women with no prenatal visits, increases in the proportion of deliveries in hospital, and with reductions in hospital case-fatality rate for delivery-associated causes. These results were robust to several model adjustments and controls for the duration and the specificity of BFP effects. While all the findings were statistical associations, the evident dose-response relationship, together with the effects on indicators of the causal pathway from exposure to the outcome—such as antenatal care and hospital deliveries—suggest a causal interpretation of these results.

The published evidence of the impact of conditional cash transfer on maternal deaths in LMICs is still limited and controversial. A study on the impact of India’s Janani Suraksha Yojana (JSY), a cash transfer programme, found no significant reduction on maternal death [6], while other reports of the Mexican CCT Oportunidades suggest some positive effects [25]. Financial incentives had also shown effects on the increase of deliveries with skilled attendants in Nepal [26]. One explanation for such different results could be the time between policy implementation and evaluation, considering that for example, JSY was evaluated 2 years after being launched [6]. As a matter of facts, there is evidence that CCT has long-term effects on education and socioeconomic outcomes [27], so analyses of CCT impacts with a long-term perspective, as we did in our study, are fundamental for a comprehensive impact evaluation of these programmes.

**Fig. 1:**
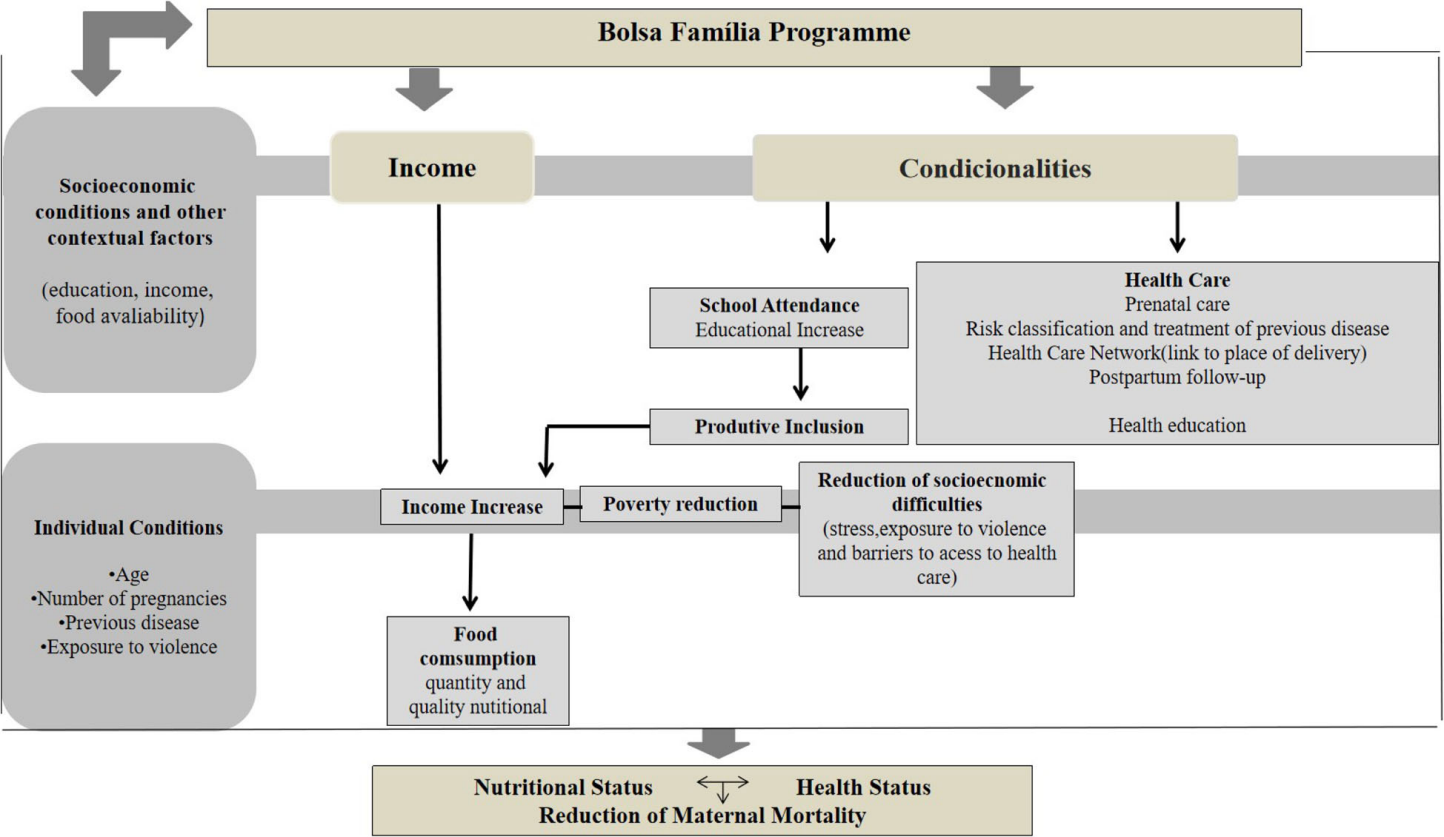
Mechanisms linking the Bolsa Familia Programme to the reduction of maternal mortality

A conditional cash transfer can impact maternal mortality through a diversity of mechanisms (Fig. 1). First, CCT conditionalities—conditions that the beneficiary must comply with in order to receive CCT money allowances—impose a minimum usage of health services for child and maternal health. As a matter of fact, the majority of CCT—including the BFP—comprise among their conditionalities the compliance with the national scheme of prenatal care visits, possibly increasing the use of services for skilled birth attendance [4, 7]. Second, money allowances could improve women nutrition and consequently their health status at the moment of delivery [5]. Third, increased demand for health services may, in the long term, also trigger improvements in the supply of services, as observed in the CCT Oportunidades [25].

In Brazil, the leading direct causes of maternal deaths are hypertensive disorders (23% of all maternal deaths), followed by sepsis (10%), haemorrhage (8%), complications of abortion (8%), placental disorders (5%), other complications of labour (4%), embolism (4%), abnormal uterine contractions (4%) and HIV/AIDS-related disorders (4%) [28]. The potential impact of cash transfer on socioeconomic factors (women empowerment in house and society, educational and economic status of women), access to facilities (distance, transportation) and availability of the quality of care (staff and equipment in health centres) could directly improve maternal nutritional status (supplementation of multiple micronutrients, folic acid and iron-folic acid during pregnancy and calcium supplementation in pregnant women with low/inadequate intake), reducing anaemia and its complications during pregnancy and birth. The programme—facilitating health care access—may impact treatment, management and referral of maternal diabetes and antihypertensive for mild to moderate hypertension, management and screening of HIV and other sexually transmitted infections, improving quality of antenatal care (at least seven visits of focused antenatal care) [28].

Another important finding is that the effect of BFP was observed after some years of exposure. This could be potentially associated with the time necessary to induce behaviour changes, as conditional cash transfer programmes rely on behaviour changes regarding maternal lifestyles and the use of health services [4]. Studies have suggested that women with larger exposure time to a CCT programme engage in higher maternal health service utilisation [29]. CCT has also shown important effects on schooling accumulation and health when maintained over the years [27, 30]. Higher schooling during childhood and adolescence, due to CCT conditionalities on school attendance, might lead to more knowledge about the importance of prenatal visits, contraceptive methods, and family planning, affecting pregnancy and fecundity [28]. Oportunidades, the Mexican CCT, has shown effect on contraceptive use and birth spacing among female household heads receiving the transfer for a long time of the programme [25]. On the other hand, by providing females with resources, CCT may change bargaining power within the household. Regarding long-term nutrition effects, a study has shown that the major period of exposure to monetary transfer by BFP tends to increase the possibility of improvements in the nutritional status of the beneficiary children and, consequently, young mothers [31].

Long-term effects that directly affect the life of the subsequent generation, such as the newborns of mothers exposed during childhood and adolescence to CCT, could also contribute to break the inter-generational cycle of poverty and poverty-associated outcomes, which is one of the main objectives of CCT since their conception [30]. In this sense, this is one of the first studies—to our knowledge—that suggest such direct effects from the exposition to CCT during childhood and adolescence to the prevention of an important health risk condition—being motherless—in the next generation of children.

One of the limitations of this study is that the fixed-effects specification of our regression models can control for unobserved variables only if they are time-constant, such as geographic, infrastructural and cultural characteristics of the municipality, but not if they are time-variant. Therefore, we have included all relevant demographic and socioeconomic confounding variables of the association between BFP and maternal mortality in the regression and adopted the strategy of fitting the model with a control outcome (under-five mortality due to accidents), showing that the effects were outcome specific. Moreover, the use of a time dummy variable allowed to adjust not only for secular trends but also for changes in specific years, such as the intensification of active research of maternal deaths by the Maternal Mortality Committee (*Comite de Mortalidade Materna)* in Brazil in the year 2009 [28]. Another limitation was selecting the municipalities with the higher quality of vital information, which strengthened the internal validity of the study but could have limited its generalizability. However, this has been a common strategy of previous nationwide impact evaluations using the same data and analytic design [8, 23], and we have evaluated that all regions of the country were represented in the cohort of studied municipalities.

One of the main strengths of the study was that we fitted the same models with two different control outcomes: the first control—U5MR from all causes—allowed us to demonstrate that the absence of effects with short BFP duration was outcome-specific and that the same models were able to reproduce the immediate BFP effects on U5MR shown in previous studies [8, 23]. The second control outcome—U5MR for accidents—demonstrated that our models were correctly showing no effects on such mortality causes, confirming findings from the literature [8].

## Conclusions

The results of our study show that high and sustained coverage of conditional cash transfers can have long-term effects on maternal mortality in vulnerable populations, potentially contributing to the reduction of health inequalities and to the achievement of the Sustainable Development Goal 3.1. For such reasons, it should be paramount that CCT are preserved during the current global economic recession due to the COVID-19 pandemic.

## Supplementary Information

**Additional file 1.** Additional Analyses and Sensivity Analyses.

## Data Availability

The data used are public and available from the Brazilian Ministry Health (DATASUS), Brazilian Statistics Institute (IBGE) and Ministry of Social Development websites: http://www2.datasus.gov.br/DATASUS/index.php, https://aplicacoes.mds.gov.br/sagi/vis/data3/data-explorer.php.

## Abbreviations

BFP: Bolsa Familia Programme
CCT: Conditional cash transfer
FE: Fixed effects
FHP: Family Health Programme
IBGE: Brazilian Institute of Geography and Statistics
LMIC: Low- and middle-income countries
