# Supplementary material for: Long-term impact of a conditional cash transfer programme on maternal mortality: a nationwide analysis of Brazilian longitudinal data
Source: BMC Med. 2021 Jun 1;19:127. doi: 10.1186/s12916-021-01994-7 (PMC8166529; doi:10.1186/s12916-021-01994-7)
Supplement: Supplementary file 1 — Additional file 1. Additional Analyses and Sensivity Analyses. [file 12916_2021_1994_MOESM1_ESM.doc]

**Additional File 1**

**Supplement to: Long-term impact of a conditional cash transfer programme on maternal mortality: a nationwide analysis of Brazilian longitudinal data**

Davide Rasella, PhD

Flávia Jôse Oliveira Alves, MsC

Poliana Rebouças, PhD

Gabriela Santos de Jesus, MsC

Maurício L. Barreto, PhD

Tereza Campello, PhD

Enny S. Paixao, PhD

1. **Duration effect of the Bolsa Familia Coverage for the complete range of years of the study**

As described in the main text, in order to evaluate BFP duration effects we used the average municipal coverage of the last n years (from 1 to the maximum allowed by the study period, 11), an indicator which captures the intensity of the intervention coverage along the previous years.^1,2^ Each average BFP municipal coverage of the last n years was categorized in quartiles representing the level of implementation, low (1st quartile), intermediate (2nd), high (3rd) and consolidated (4th), as in previous studies.^2^ This coverage indicator was categorized as well, for comparability reasons with BFP and other studies,^1,2^ in quartiles. Table S1 show BFP coefficients (expressed as rate ratios) for all 11 possible years of duration coverage in the study period. While there are fluctuations and not always clear dose-response associations with BFP coverage in some years - when the mortality fluctuations shown above occur - the estimates from the initial and final years of the period are showing a clearer duration effect.


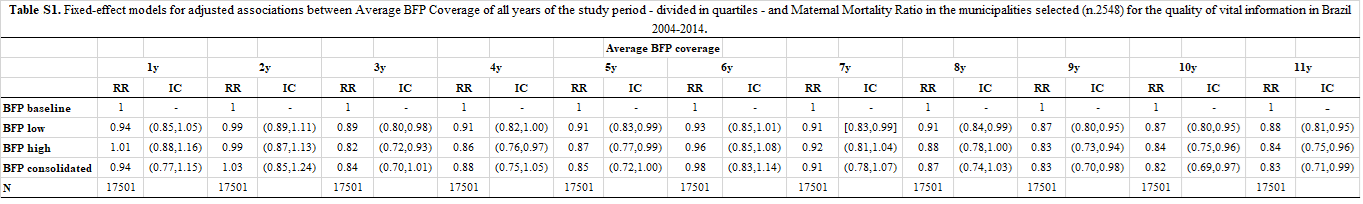


1. **Maternal Mortality evolution in Brazil and the time variable in the model**

The time variable has been included in the regression model as a dummy variable, using as a baseline the first year of the study period. The choice of using a dummy variable instead of a continuous (or dichotomous as the other covariates) variable was due to the influence of events which hit only in specific and isolated years almost all municipalities, for example the H1N1 outbreak in 2009 ^3^ and the increased activities to improve the reporting of maternal deaths, including compulsory investigations into deaths of women of reproductive age, with the increase of maternal mortality committees to all 27 Brazilian states in the year 2009 and 2010 (Figure S1).^3,4^  Below there are trends to maternal mortality along the period of the study based on information of DATASUS to all Brazilian municipalities, selected municipalities with adequate information, publications about the 30 years of National Health^5^ and information by World Health Organization (WHO).^6^

**Figure S1. Maternal mortality ratios along the period under study (2004-2014), Brazil.**


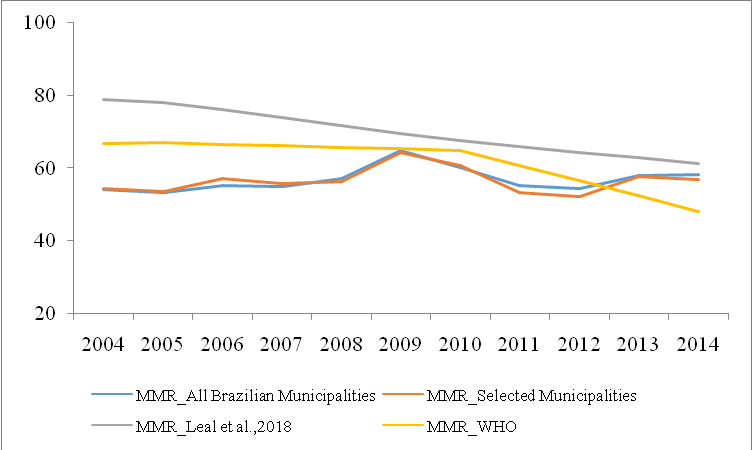


In Table S2 we show the outputs of the same models of Table 2 in the main text, but with the values of the time dummy variable for all years. As expected, the models were able to adjust for the peak in Maternal Mortality of the years 2008 and 2009 with time variable coefficients highly positive during the period.

| **Table S2**. **Fixed-effect models for adjusted associations between Average BFP Coverage of the last 1,2,5,10 and 11 years - divided in quartiles - and Maternal Mortality Ratio in the municipalities selected (n.2,548) for the quality of vital information in Brazil 2004-2014.** | | | | | | | | | | |
| --- | --- | --- | --- | --- | --- | --- | --- | --- | --- | --- |
|  | Average BFP cov 1 year | | Average BFP cov 2 year | | Average BFP cov 5 year | | Average BFP cov 10 year | | Average BFP cov 11 year | |
|  | RR | IC | RR | IC | RR | IC | RR | IC | RR | IC |
| BFPcov 1q | 1.00 | _ | 1.00 | _ | 1.00 | _ | 1.00 | _ | 1.00 | _ |
| BFP cov 2q | 0.94 | (0.85,1.05) | 0.99 | (0.89,1.11) | 0.91 | (0.83,0.99) | 0.87 | (0.80, 0.95) | 0.88 | (0.81,0.95) |
| BFP cov 3q | 1.01 | (0.88,1.16) | 0.99 | (0.87,1.13) | 0.87 | (0.77,0.99) | 0.84 | (0.75,0.96) | 0.84 | (0.75,0.96) |
| BFP cov 4q | 0.94 | (0.77,1.15) | 1.03 | (0.85,1.24) | 0,85 | (0.72,1.00) | 0.82 | (0.69,0.97) | 0.83 | (0.71,0.99) |
| FHP cov 1q | 1.00 | _ | 1.00 | _ | 1.00 | _ | 1.00 | _ | 1.00 | _ |
| FHP cov 2q | 0.97 | (0.90,1.06) | 0.97 | (0.89,1.06) | 0.99 | (0.91,1.08) | 0.99 | (0.91,1.08) | 0.99 | (0.91,1.08) |
| FHP cov 3q | 0.97 | (0.86,1.10) | 0.96 | (0.84,1.09) | 1.00 | (0.88,1.14) | 0.99 | (0.87,1.13) | 0.99 | (0.86,1.13) |
| FHP cov 4q | 0.93 | (0.79,1.08) | 0.91 | (0.78,1.07) | 0.95 | (0.81,1.12) | 0.94 | (0.79,1.11) | 0.93 | (0.79,1.10) |
| Income percapita | 0.84 | (0.72,0.98) | 0.84 | (0.72,0.98) | 0.85 | (0.72,0.99) | 0.85 | (0.72,0.99) | 0.85 | (0.72,0.99) |
| Poverty rate | 1.01 | (0.91,1.12) | 0.99 | (0.89,1.09) | 0.97 | (0.88,1.08) | 0.98 | (0.88,1.09) | 0.98 | (0.89,1.09) |
| Illiteracy rate | 0.96 | (0.81,1.13) | 0.96 | (0.81,1.13) | 0.95 | (0.81,1.12) | 0.95 | (0.81,1.13) | 0.95 | (0.81,1.13) |
| Piped Water | 1.04 | (0.93,1.16) | 1.04 | (0.93,1.16) | 1.04 | (0.93,1.16) | 1.04 | (0.93,1.16) | 1.04 | (0.93,1.16) |
| Phisicians/hab | 1.01 | (0.91,1.11) | 1.01 | (0.91,1.11) | 1.00 | (0.91,1.11) | 1.00 | (0.91,1.10) | 1.00 | (0.91,1.10) |
| Private care coverage | 0.99 | (0.84,1.17) | 0.99 | (0.84,1.17) | 0.99 | (0.84,1.17) | 0.99 | (0.84,1.17) | 0.99 | (0.84,1.17) |
| Hospitalization rates | 0.91 | (0.71,1.15) | 0.91 | (0.71,1.16) | 0.91 | (0.72,1.16) | 0.91 | (0.72,1.16) | 0.91 | (0.72,1.16) |
| Year 2005 | 1.00 | (0.90,1.11) | 0.99 | (0.89,1.11) | 1.01 | (0.91,1.12) | 1.01 | (0.91,1.13) | 1.01 | (0.91,1.13) |
| Year 2006 | 1.08 | (0.96,1.21) | 1.06 | (0.94,1.20) | 1.12 | (1.00,1.25) | 1.12 | (1.00,1.25) | 1.12 | (1.00,1.25) |
| Year 2007 | 1.06 | (0.94,1.19) | 1.05 | (0.92,1.19) | 1.12 | (0.98,1.26) | 1.13 | (1.00,1.28) | 1.13 | (1.00,1.28) |
| Year 2008 | 1.08 | (0.96,1.22) | 1.07 | (0.95,1.22) | 1.15 | (1.01,1.32) | 1.18 | (1.03,1.34) | 1.18 | (1.03,1.34) |
| Year 2009 | 1.25 | (1.10,1.42) | 1.24 | (1.09,1.42) | 1.33 | (1.16,1.53) | 1.37 | (1.19,1.57) | 1.37 | (1.19,1.57) |
| Year 2010 | 1.18 | (1.04,1.35) | 1.17 | (1.02,1.34) | 1.26 | (1.09,1.44) | 1.30 | (1.13,1.50) | 1.30 | (1.13,1.50) |
| Year 2011 | 1.04 | (0.91,1.18) | 1.03 | (0.90,1.18) | 1.10 | (0.96,1.27) | 1.16 | (1.00,1.34) | 1.15 | (1.00,1.33) |
| Year 2012 | 1.01 | (0.89,1.16) | 1.00 | (0.88,1.15) | 1.08 | (0.94,1.24) | 1.17 | (1.00,1.36) | 1.16 | (0.99,1.36) |
| Year 2013 | 1.12 | (0.98,1.27) | 1.11 | (0.96,1.27) | 1.20 | (1.04,1.38) | 1.29 | (1.10,1.51) | 1.28 | (1.09,1.50) |
| Year 2014 | 1.10 | (0.97,1.25) | 1.09 | (0.97,1.25) | 1.08 | (0.94,1.24) | 1.17 | (1.00,1.36) | 1.16 | (0.99,1.36) |
| Number of observations | 17501 |  | 17501 |  | 17501 |  | 17501 |  | 17501 |  |

1. **Negative binomial regression models with fixed effect specifications in impact evaluations**

Negative binomial (NB) regression models are used when the outcome to be analysed is a count data and the Poisson model assumption that the mean is equal to the variance does not hold, usually because the data are *overdispersed.*^7^ The NB regression can be used with longitudinal or panel data, where the same unit of analysis has repeated observations over a period of time.^8^In this case, in addition to the disturbance or error term, panel data models include a second term to control for unobserved time-invariant characteristics of the unit of analysis, or panel. According to how this term is estimated, the models can be distinguished infixed effects or random-effects models. From a statistical point of view, the choice between fixed-effects and random-effects models is based on the Hausman specification test.^7^ In impact evaluations fixed-effects (FE) models are usually preferred because they permit correlations between the unobserved time-invariant term and the explanatory variables.^7^ In our case the time-invariant term could represent unobserved characteristics of the municipality such as geographical, historical, socio-cultural or socio-economic characteristics that did not change during the period of the study. In fixed effects models, but not in random ones, those characteristics could be correlated with the treatment variables, such as the BFP or FHP coverage. If for example these interventions were implemented with priority in remote and poor areas with higher mortality rates, and variables linked to those characteristics were not included in the model, the estimates of the intervention effects could suffer from selection bias. Fixed effects models allow controlling for this selection bias because the fixed effect term of the equation represents these unobserved time-invariant characteristics of the panel.^9^

1. **The Regression Model**

The regression model used in the equation can be shown as:

*log*(*MMR_it_)= α_i_ + β_q1-4_*BFP*_q1-4n it_ + β_k_ x_k it_ + β_t_ t_t_ + ε_it_*

Where:

*t* refers to the year (from 2004 to 2014) and *i* refers to an individual municipality;

*MMR_it_* is the Maternal Mortality Ratio in municipality *i* in year *t*;

BFP*_q1-4n it_*is the average municipal BFP coverage of the last *n* years categorized in quartiles and introduced in the regression as dummy variable in municipality *i* in year *t* with a coefficient of *βq1*(lower quartile, the baseline coverage, per definition always equal to 1),*βq2* (2^nd^ quartile, defined as low municipal coverage), *βq3* (3^rd^ quartile, defined as intermediate municipal coverage), *βq4* (4^th^ quartile, defined as high municipal coverage).

*x_k it_*refers to each covariate (*k*) for municipality *i* in year *t* with coefficient *βk*;

*t_t_* refers to the time-variable expressed as dummy with coefficient *β_t_*in year *t*

*_ai_* is the fixed effect (municipality-level time-invariant effect) error term (not estimated);

*ε_it_* is the idiosyncratic error term for municipality i in year t (not estimated);

Using an off-set model we obtain a rate ratio (RR). For example, in the model that consider the BFP duration of 10 years, a value of RR 0.84 for BFP high municipal coverage refers to a ratio between the mortality rates of the lowest and the highest quartile of the average municipal BFP coverage of the last 10 years and is thus interpreted as an associated 16% reduction in maternal mortality. Because this is a fixed-effect longitudinal regression, we only associate this to changes within the municipality (i.e. changes in within-municipality mortality and within-municipality BFP coverage). Between-municipality effects (i.e. difference between municipalities) are not estimated.

1. **Sensitivity Analyses**

A wide range of sensitivity analyses was performed to test the robustness of our findings.

1. Lag Effects

Lag effects are often used to show how exposure in previous years could affect the outcomes in later years and is based on the modelling of the association between exposure variable of the previous *i-n* years with the outcome variables of the *i* year. While this is a powerful tool for analytic evaluations, unless the outcome has specific periods of latency (for example an infectious disease), for public intervention evaluations it is difficult to assume an a priori specific lag effect. A lag analysis was excluded as the main method to evaluate the BFP long-term effects for two reasons: one associated with statistical issues and the other to theoretical aspects.

Regarding the statistical issue, in our case, the main drawback of the lag approach is that it reduces of *n* years the period of the study. As a matter of fact we cannot include in the study period years before 2004 because cash transfer programs such as *Bolsa Escola, Bolsa Alimentacao* and *Vale gas* – among others- were attending the majority of the families who received, from 2004, BFP, but no information on the coverage of these programs is available. The lost of study years in our lag analyses (dropping the number of observations from 17501 up to 2220) could explain the loss of stability and statistical significance of the estimates shown in Table S3.

**Table S3. Fixed-effect models for adjusted associations between lag BFP Coverage - divided in quartiles - and Maternal Mortality Ratio in the municipalities selected (n.2548) for the quality of vital information in Brazil 2004-2014.**

|  | Average 1 year BFP coverage | | | | |  | Average 2 year BFP coverage | | | | |
| --- | --- | --- | --- | --- | --- | --- | --- | --- | --- | --- | --- |
|  | 7y Lag | |  | 8y Lag | |  | 7y Lag | |  | 8y Lag | |
|  | RR | IC95% |  | RR | IC95% |  | RR | IC95% |  | RR | IC95% |
| BFP Quartile 1 | 1.00 | - |  | 1.00 | - |  | 1.00 | - |  | 1.00 | - |
| BFP Quartile 2 | 1.05 | (0.89,1.23) |  | 1.03 | (0.86,1.25) |  | 0.97 | (0.80,0.98) |  | 0.96 | (0.82,1.00) |
| BFP Quartile 3 | 0.92 | (0.73,1.15) |  | 1.01 | (0.77,1.33) |  | 0.97 | (0.72,0.93) |  | 0.91 | (0.76,0.97) |
| BFP Quartile 4 | 0.96 | (0.68,1.35) |  | 0.94 | (0.62,1.41) |  | 1.02 | (0.70,1.01) |  | 0.98 | (0.75,1.05) |
| Number of observations | 17501 |  |  | 17501 |  |  | 17501 |  |  | 17501 |  |

From a theoretical point of view the lag analysis assumes that a *i-n* year exposure has an exact corresponding effect on the *i* year outcome, which is hard to justify for a complex intervention such as BFP and a composite outcome such as maternal mortality. A duration analysis, which consider BFP exposure in the model not only as the coverage of the *i* year but as the result of the consolidation of BFP coverage of the previous *n* years ^1^ could be more adequate to evaluate long-term effects of articulated poverty-relief interventions such as cash transfer programmes.

1. Poisson vs Negative Binomial models

Poisson models were fitter as sensitivity analyses providing similar estimates (Table S4). Negative binomial models were preferred because they better fit overdispersed count data, as explained above, and because had slightly lower Akaike Information Criteria (AIC), while maintaining almost identical effect estimates (Table S4).

| **Table S4. Fixed-effect models for adjusted associations between lag BFP Coverage - divided in quartiles - and Maternal Mortality Ratio in the municipalities selected (n.2548) for the quality of vital information in Brazil 2004-2014.** | | | | | | | | | | | |
| --- | --- | --- | --- | --- | --- | --- | --- | --- | --- | --- | --- |
|  | Average BFP cov 1 year | | Average BFP cov 2 year | | Average BFP cov 5 year | | Average BFP cov 10 year | | | Average BFP cov 11 year | |
|  | RR | 95% CI | RR | 95% CI | RR | 95% CI | RR | 95% CI | RR | | 95% CI |
| **Poisson** |  |  |  |  |  |  |  |  |  | |  |
| BFP quart 1 | 1,00 | - | 1,00 | - | 1,00 | - | 1,00 | - | 1,00 | | - |
| BFP quart 2 | 0.94 | (0.85,1.05) | 0.99 | (0.89,1.10) | 0.91 | (0.82,0.99) | 0.87 | (0.80,0.95) | 0.88 | | (0.81, 0.95) |
| BFP quart 3 | 1.01 | (0.87,1.12) | 0.99 | (0.77,0.99) | 0.87 | (0.75,0.96) | 0.85 | (0.75,0.96) | 0.84 | | (0.87,1.16) |
| BFP quart 4 | 0.92 | (0.76,1.14) | 1.02 | (0.84,1.24) | 0.85 | (0.72,1.00) | 0.82 | (0.70,0.97) | 0.84 | | (0.71,0.99) |
| Number of observations | 17501 |  | 17501 |  | 17501 |  | 17501 |  | 17501 | |  |
| AIC | 16259.97 |  | 16263.67 |  | 16258.11 |  | 16252.46 |  | 16253.54 | |  |
| **Neg Binomial** |  |  |  |  |  |  |  |  |  | |  |
| BFP quart 1 | 1,00 | - | 1,00 | - | 1,00 | - | 1,00 | - | 1,00 | | - |
| BFP quart 2 | 0.94 | (0.89,1.11) | 0.99 | (0.82,0.99) | 0.91 | (0.80,0.95) | 0.87 | (0.81,0.95) | 0.88 | | (0.85,1.05) |
| BFP quart 3 | 1.01 | (0.88,1.16) | 0.99 | (0.87,1.12) | 0.87 | (0.77,0.99) | 0.84 | (0.75,0.96) | 0.84 | | (0.75,0.96) |
| BFP quart 4 | 0.94 | (0.85,1.24) | 1.02 | (0.72,1.00) | 0.85 | (0.69,0.97) | 0.82 | (0.71,0.99) | 0.82 | | (0.77,1.15) |
| Number of observations | 17501 |  | 17501 |  | 17501 |  | 17501 |  | 17501 | |  |
| AIC | 16259.25 |  | 16262.72 |  | 16257.40 |  | 16252.66 |  | 16252.74 | |  |

1. Different Categorizations

To verify if the choice of the number of categories – different from the one chosen based on previous studies ^2^ - affected the results, we fitted the same models with different categorizations of BFP coverage obtaining similar findings (Table S5).

**Table S5. Fixed-effect Poisson and Negative Binomial models for adjusted associations between lag BFP Coverage - divided in quartiles- and Maternal Mortality Ratio in the municipalities selected (n.2548) for the quality of vital information in Brazil 2004-2014.**

|  | Average BFP cov 1 year | | Average BFP cov 2 year | | Average BFP cov 5 year | | Average BFP cov 10 year | | Average BFP cov 11 year | |
| --- | --- | --- | --- | --- | --- | --- | --- | --- | --- | --- |
|  | RR | 95% CI | RR | 95% CI | RR | 95% CI | RR | 95% CI | RR | 95% CI |
| BFP Quartile 1 | 1.00 |  | 1.00 |  | 1.00 |  | 1.00 |  | 1.00 |  |
| BFP Quartile 2 | 0.94 | (0.85,1.05) | 0.99 | (0.89,1.11) | 0.91 | (0.82,0.99) | 0.87 | (0.80,0.95) | 0.88 | (0.81,0.95) |
| BFP Quartile 3 | 1.01 | (0.87,1.12) | 0.99 | (0.77,0.99) | 0.87 | (0.75,0.96) | 0.84 | (0.75,0.96) | 0.84 | (0.88,1.16) |
| BFP Quartile 4 | 0.94 | 0.77,1.15) | 1.02 | (0.85,1.24) | 0.85 | (0.72,1.00) | 0.82 | (0.69,0.97) | 0.82 | (0.71,0.99) |
| Number of observations | 17501 |  | 17501 |  | 17501 |  | 17501 |  | 17501 |  |
| BFP Quintile 1 | 1.00 |  | 1.00 |  | 1.00 |  | 1.00 |  | 1.00 |  |
| BFP Quintile 2 | 0.94 | (0.84,1.05) | 0.99 | (0.90,1.10) | 0.95 | (0.87,1,04) | 0.84 | (0.77, 0.92) | 0.85 | (0.78,0.92) |
| BFP Quintile 3 | 1.01 | (0.88,1.15) | 0.92 | (0.81,1.04) | 0.87 | (0.77,0.99) | 0.82 | (0.72,0.94) | 0.85 | (0.75,0.96) |
| BFP Quintile 4 | 0.99 | (0.84,1.14) | 0.98 | (0.80,1.08) | 0.92 | (0.62,0.65) | 0.73 | (0.65,0.89) | 0.76 | (0.84,1.17) |
| BFP Quintile 5 | 0.96 | (0.77,1.19) | 0.95 | (0.78,1.17) | 0.90 | (0.74,1.09) | 0.69 | (0.56,0.84) | 0.73 | (0.59,0.89) |
| Number of observations | 17501 |  | 17501 |  | 17501 |  | 17501 |  | 17501 |  |
| BFP Sextile 1 | 1.00 |  | 1.00 |  | 1.00 |  | 1.00 |  | 1.00 |  |
| BFP Sextile2 | 1.02 | (0.92,1.12) | 0.95 | (0.86,1.05) | 0.96 | (0.88,1.05) | 0.87 | (0.79,0.96) | 0.87 | (0.79,0.96) |
| BFP Sextile 3 | 1.00 | (0.88,1.15) | 0.94 | (0.82,1.07) | 0.87 | (0.76,0.98) | 0.84 | (0.74,0.96) | 0.82 | (0.72,0.94) |
| BFP Sextile 4 | 1.06 | (0.91, 1.22) | 0.96 | (0.82,1.11) | 0.85 | (0.72,0.98) | 0.80 | (0.68,0.94) | 0.78 | (0.67,0.92) |
| BFP Sextile 5 | 1.12 | (0.93,1.35) | 0.88 | (0.74,1.04) | 0.85 | (0.72,1.01) | 0.80 | (0.66,0.97) | 0.78 | (0.64,0.95) |
| BFP Sextile 6 | 1.02 | (0.82,1.31) | 0.86 | (0.69,1.07) | 0.87 | (0.70,1.08) | 0.72 | (0.56,0.91) | 0.71 | (0.56,0.90) |
| Number of observations | 17501 |  | 17501 |  | 17501 |  | 17501 |  | 17501 |  |

1. **Estimating the percentage of maternal deaths from vulnerable segments of the population**

As explained in the previous studies,^2^ impact evaluations with ecologic design can not test if the individuals exposed to the intervention are the ones which are responsible for the improvements in health outcomes we measure in the municipality. Despite this being an important limitation for the majority of these studies, for specific targeted interventions – such as poverty-relief programs – and specific poverty-associated mortalities – such as maternal mortality ^10^- we can reasonably assume that almost all mortality improvements in the municipality are coming from the poorest individuals, that have also been exposed to the targeted intervention. As a matter of fact, if we consider the unit of analysis of an ecological study, for example, a municipality, divided into two different population groups with different mortality rates (MR), with MR_p_ being the MR of the poorest part of the population, and MR_r_ being the MR of the rest of the population, the Rate Ratio (RR) is: RR= MR_p_ / MR_r_.

If we consider the deaths from the poorest group (D_p_) over the population of the poorest group (P_p_) and the deaths from the rest of the population (D_r_) over the rest of the population (P_r_), We can obtain the total deaths in the county (D_tot_) and the total population (P_tot_) from the following equations:

MR_p_ = RR × MR_r_

D_p_ / P_p_ = RR × D_r_ / P_r_

D_p_ = RR × (D_tot_ - D_p_) × P_p_ / P_r_

D_p_ × ( 1 + RR × P_p_ / P_r_ ) = RR × P_p_ × D_tot_ / P_r_

D_p_ = RR × P_p_ × D_tot_ / [ P_p_ × (RR-1) + P_tot_ ]

D_p_ = K_p_ × D_tot_

K_p_ represents the proportion of deaths that come from the poorest segment of the county’s population, and depends on the Mortality Rate Ratio between the two population groups and the proportion of poor people over the total population of the county (PP_p_) according to the following equation:

K_p_ = RR × PP_p_ / [ PP_p_ × (RR-1) + 1 ]

Considering different values of Mortality Rate Ratios, the proportion of deaths that come from the poorest part of the population has a curvilinear relationship with the proportion of poor people in the county (Figure S2). If we consider an under-five mortality rate ratio (RR) of 2.3,7 in a county with 30% poor people the proportion of under-five deaths attributable to them will be 50%. In the case of segments of the population in extreme poverty the RR, and consequently the proportion of deaths attributable to them, will be considerably higher. The RR for specific causes,^8^ especially poverty-related causes such as maternal mortality, can be so high that the deaths attributable to extremely poor people reach almost the totality of the deaths for this specific cause in the county, as shown in the figure.

**Figure S2: Proportion of deaths coming from the poorest part of the population (K_p_) as function of the proportion of poor people in the county (PP_p_) and according to different values of mortality rate ratio (RR).**

**References**

1. Rasella, D., Harhay, M. O., Pamponet, M. L., Aquino, R. & Barreto, M. L. Impact of primary health care on mortality from heart and cerebrovascular diseases in Brazil: a nationwide analysis of longitudinal data. *BMJ* 349, g4014 (2014).

2. Rasella, D., Aquino, R., Santos, C. A. T., Paes-Sousa, R. & Barreto, M. L. Effect of a conditional cash transfer programme on childhood mortality: a nationwide analysis of Brazilian municipalities. *Lancet Lond. Engl.*382, 57–64 (2013).

3. Brasil. Ministério da Saúde. Secretaria de Vigilância em Saúde. Departamento de Vigilância de Doenças e Agravos não Transmissíveis e Promoção da Saúde. Saúde Brasil 2017 : uma análise da situação de saúde e os desafios para o alcance dos objetivos de desenvolvimento sustentável [recurso eletrônico] / Ministério da Saúde, Secretaria de Vigilância em Saúde, Departamento de Vigilância de Doenças e Agravos não Transmissíveis e Promoção da Saúde. – Brasília : Ministério da Saúde, 2018.

4. Victora, C. G., Aquino, E. M., do Carmo Leal, M., Monteiro, C. A., Barros, F. C., & Szwarcwald, C. L. (2011). Maternal and child health in Brazil: progress and challenges. *The Lancet*, *377*(9780), 1863-1876.

5. Leal, Maria do Carmo, et al. "Saúde reprodutiva, materna, neonatal e infantil nos 30 anos do Sistema Único de Saúde (SUS)." *Ciência & Saúde Coletiva* 23 (2018): 1915-1928.

6. World Health Organization. Trends in maternal mortality: 1990-2015: estimates from WHO, UNICEF, UNFPA, World Bank Group and the United Nations Population Division: executive summary. No. WHO/RHR/15.23. World Health Organization, 2015.

7. Hilbe, J. M. Negative Binomial Regression by Joseph M. Hilbe. *Cambridge Core* (2011). doi:10.1017/CBO9780511973420

8. Wooldridge, J. M. *Introductory Econometrics: A Modern Approach*. (South-Western Pub, 2012).

9. Khandker, S. R., Koolwal, G. B. & Samad, H. A. *Handbook on impact evaluation: quantitative methods and practices*. (World Bank, 2010).

10. Ronsmans, C. & Graham, W. J. Maternal mortality: who, when, where, and why. *The Lancet.* 368, 1189–1200 (2006).
